# Optimal Function Prediction of Key Aberrant Genes in Early-onset Preeclampsia Using a Modified Network-based Guilt by Association Method

**Published:** 2018-11

**Authors:** Jing WANG, Yanping BI, Junxia LI, Yanfang TIAN, Xue YANG, Zhongfang SUN

**Affiliations:** 1. Dept. of Obstetrics, Seventh People’s Hospital of Jinan, Jiyang, Shandong, China; 2. Dept. General Surgery, Seventh People’s Hospital of Jinan, Jiyang, Shandong, China

**Keywords:** Early-onset preeclampsia, Optional function, Co-expressed genes network, Multifunctionality algorithm

## Abstract

**Background:**

To predict the optimal functions of key aberrant genes in early-onset preeclampsia (EOPE) by using a modified network-based gene function inference method.

**Methods:**

First, differentially expressed genes (DEGs) were extracted using linear models for microarray data (LIMMA) package. Then the Spearman’s rank correlation coefficient was calculated to assess co-expressed strength of each interaction between DEGs, based on which the co-expressed genes network was constructed to vividly exhibit their interlinking relationship. Subsequently, Gene ontology (GO) annotations for EOPE were collected according to known confirmed database and DEGs. Ultimately, the multifunctionality algorithm was used to extend the “guilt by association” method based on the co-expressed network, and a 3-fold cross validation was operated to evaluate the accuracy of the algorithm.

**Results:**

During the process, the GO terms, of which the area under the curve (AUC) over 0.7 were screened as the optimal gene functions for EOPE. Six functions including the ion binding and cellular response to stimulus were determined as the optimal gene functions.

**Conclusion:**

Such findings should help to better understand the pathogenesis of EOPE, so as to provide some references for clinical diagnosis and treatment in the future.

## Introduction

Early-onset preeclampsia (EOPE) is a human-pregnancy-specific disease and it complicates approximately 4‰ pregnancies in nulliparous women worldwide and is a significant source of maternal and neonatal morbidity and mortality (1–3). Compared with mothers without preeclampsia, there is a 10-fold higher risk of perinatal death observed among mothers with EO-PE while a 2-fold increased risk evident with the late-onset disease (4). Most of maternal and fetal deaths are due to lack of prenatal care, inappropriate diagnosis and management of patients with EOPE. Therefore, in order to safely prolong the preterm gestation, it is necessary for us to explore the pathogenesis and make accurate and timely diagnosis.

The expression of aberrant genes has been demonstrated to play significant roles in EOPE pathogenesis (5, 6). For example, Yang et al have found osteoprotegerin gene variants is associated with early-onset severe preeclampsia. It is reported that miR-1301 is dysregulated in EOPE and could possibly play a role in the regulation of leptin during pregnancy (7). However, genes do not work in isolation but interact with each other collectively (8), and understanding the differential co-expression network will be conducive to elucidate EOPE.

In this study, we used a modified GBA method to predict the optimal function of aberrant genes in EOPE based on the differentially expressed genes (DEGs) co-expressed network. These gene functions tend to have potential for understanding the mechanisms underlying EOPE and be helpful for making early diagnosis of EOPE.

## Methods

### Gene expression in EOPE

Eight EOPE placentas (EOPE group) and 8 gestational age matched controls (control group) were subjected to bioinformatics analysis. After preprocessing and mapping between genes and probes, we got 21,036 gene expression profile data.

### Detections of DEGs

The differentially expressed values of genes in the two groups were assessed with the LIMMA procedure package which is a linear model for microarray data (9). The *P* values for all genes were amplified into -log10 form after being conducted with *t* and *F* test. Genes with threshold value of *P* < 0.02 and |log2FoldChange| >2 were regarded as DEGs.

### Construction of co-expressed network

We inputted SRCC of DEGs into the Cytoscape software (v3.5.1) to visualize their co-expression network which is an entity composed of edges and nodes. Then the topology characteristics of constructed network were analyzed based on the distribution of node degree, a measurement of connectivity in the co-expression network. Besides, the SRCC was determined as the weight value of the connective edge between co-expressed gene pairs, and the greater the weight value was, the closer the two genes relation was. Then the edges with threshold of weight values over 0.8 were selected to construct the subnetwork which could display the connected relation more vividly.

### Inferring the optimal function of network DEGs

Overall, 19,003 GO terms of 18,402 genes were downloaded from the GO database (http://geneontology.org/). Then the DEGs were enriched to the GO terms, and 65 GO terms which aggregated more than 20 DEGs were screened out to get more stable performance.

Thus, we improved gene function prediction performance in EOPE progression based on neighbor voting, including the nearest-neighbor voting and indirect connections. Concretely, for each gene i in the co-expressed network, all other neighbor genes of gene i were voted to each GO annotation K above, and the multifunctionality (MF) scores for each gene i within the K-GO term were calculated based on the following equation:

$${\text{MF}}_{\text{i}}=\sum_{\text{i}|{\text{Gene}}_{\text{i}}\in {\text{GO}}_{\text{K}}}\frac{1}{{\text{Num}}_{{\text{in}}_{\text{K}}}*{\text{Num}}_{{\text{out}}_{\text{K}}}}$$

In this formula, Num_in_K_ is the number of neighbor genes within GO term K, and Num_out_K_ is the number of neighbor genes outside GO term K. This algorithm is available at http://www.genelibs.com/gb.

Through-out, our evaluation for the algorithm’s efficacy is function-centric (10). When we use it as a ‘‘predictor’’ of GO category membership, we should get values of the area under the curve (AUCs) over 0.5 for many GO terms. In the process, the task set provides the true positives, which allows us to generate a receiver-operator characteristic (ROC) curve for each GO term in the task set, then we use the AUCs of the ROC curve as the score for that GO term, and the average AUC of those terms as the measure of their performance. Certainly, the GO terms which had the greater AUC implied well-performable and were regarded as the optional gene functions in EOPE.

## Results

### The C4orf48 and SCN9A were the key aberrant genes in co-expressed network.

Before the construction of co-expressed network, we firstly identified DEGs between EOPE and normal samples with the Limma procedure package. After the adjustment using *t* and *F* test, 81 DEGs with the thread of *P* < 0.02 and |log2FoldChange| >2 were screened out. The top 20 DEGs were found and the most significant DEGs were IER5, ITSN1, PPP1R16B, SLC16A10, and TNFAIP.

**Fig. 1:**
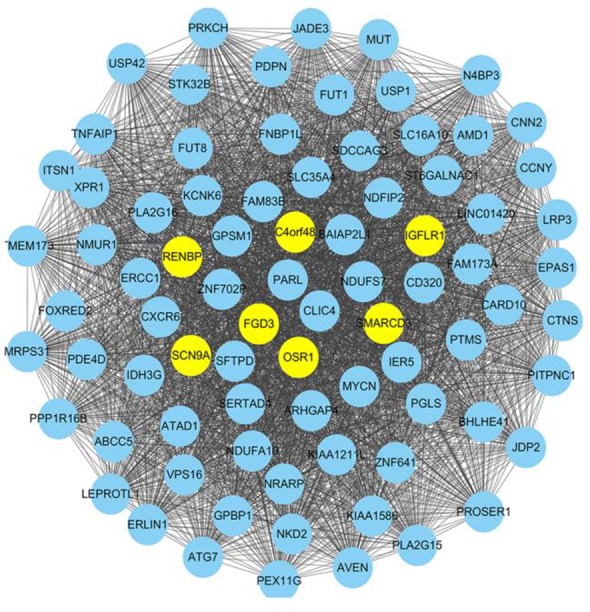
The co-expressed genes network construction for early-onset preeclampsia (EOPE). The co-expressed genes network was constructed using the Cytoscape software. There were 81 nodes and 3240 interactions, besides, FGD3, OSR1, C4orf48, SCN9A, RENBP, SMARCD3, and IGFLR1 with higher degrees were identified out

**Fig. 2:**
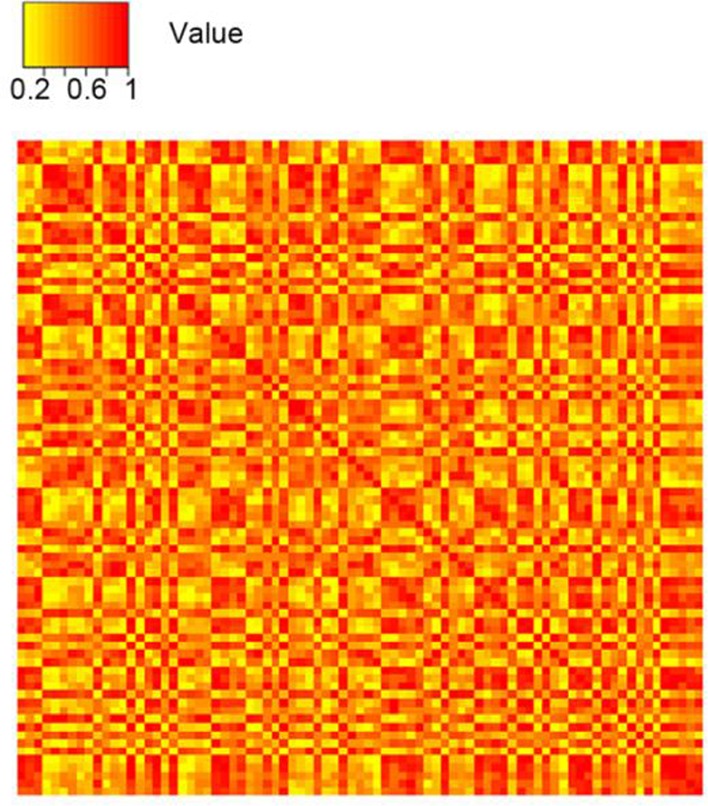
The matrix color map clarified weight distribution for each interaction. In the matrix color map, the SRCC was used to assign a weight value to an edge in the form of a colored square. The horizontal axis shows the clustering of GO term, and the vertical axis represents the clustering of the DEGs. The deeper the color was, the larger the weight value was

In order to further explore the interactions of DEGs above, the SRCC was calculated to assess co-expressed strength of each interaction between DEGs, and the co-expressed network (Fig. 1) was constructed using the Cytoscape software (v3.5.1) and the matrix color map was also plotted based on the SRCC (Fig. 2).

**Fig. 3:**
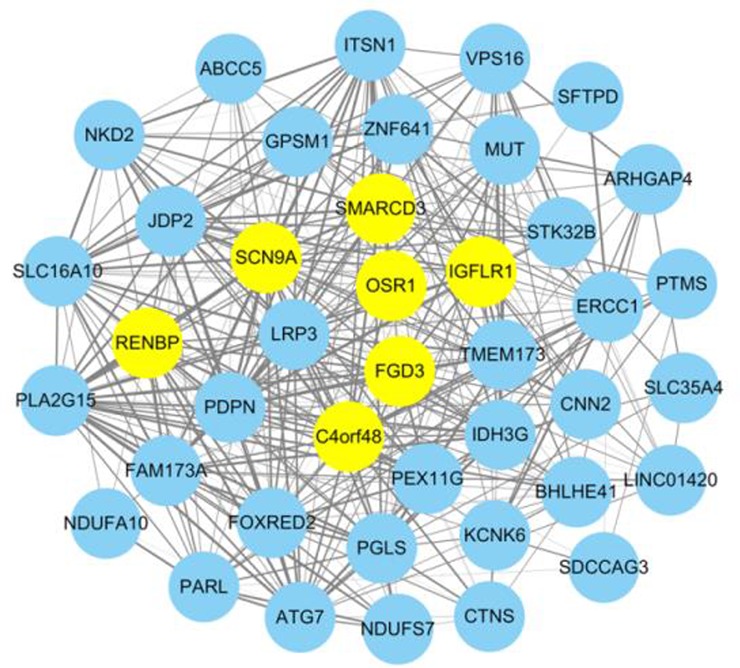
Sub-network. Sub-network using the cut-off threshold of weight value over 0.8. FGD3, OSR1, C4orf48, SCN9A, RENBP, SMARCD3, and IGFLR1 which had higher degrees in the co-expressed genes network also existed in this sub-network

In the matrix color map, the SRCC was used to assign a weight value to an edge in the form of a colored square. In this co-expressed network, there were 81 nodes and 3240 interactions. Top 7 nodes with the higher degree were marked using yellow highlights in Fig. 1. They were FGD3, OSR1, C4orf48, SCN9A, RENBP, SMARCD3, and IGFLR1, indicated that they were in a greater number of sets of genes and played more important roles in the development of EOPE. Further, to display the interacted strength between gene pairs more vividly, a sub-network with the edges weight values > 0.8 was drawn (Fig. 3). In the sub-network, there were 41 nodes and 393 interactions. Interestingly, C4orf48 and SCN9A also had the highest degree (degree=30), and the other five DEGs which had higher degree in the co-expressed network also existed in this sub-network.

### Six GO terms with AUC over 0.7 were determined as the optimal gene functions in EOPE.

**Fig. 4:**
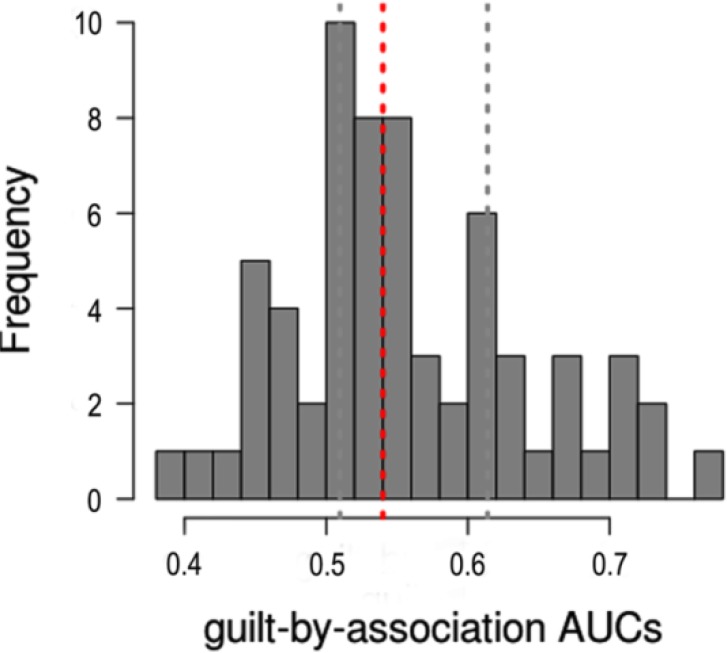
Gene function prediction performance using modified guilt by association (GBA). The histogram of AUCs across all GO terms can be obtained in the 3-fold cross validation process

We introduced the MF algorithm to improve the classifiers’ performance in gene function prediction involved in EOPE. To evaluate the algorithm’s efficacy, the 3-fold cross validation was performed using the gene sets based on MF. The AUC distribution for GO terms was shown in Fig. 4. The AUCs for the majority of functional terms were over 0.5, especially, some GO terms for which performance was quite good (over 0.7). Further, Six GO terms were screened out as the optimal gene functions.

## Discussion

The large-scale analysis of biological information was the symbol of post-genome era, especially, in which the functional genomics is one of the hotspots. It is of great significance to predict the function of genomic elements for us to reveal the origin and evolution of life, and to excavate pathogenesis of diseases. Modeling process occurring in living organisms is one of the main goals in bioinformatics, and gene networks have become one of the most important approaches to discover which gene-gene relationships are involved in a specific biological process (11, 12).

In this study, we successfully predicted the optional function of key aberrant genes by gene networks for EOPE. Concretely, firstly, the 81 DEGs were screened out with the Limma procedure package, and the most significant DEGs were IER5, ITSN1, PPP1R16B, SLC16A10, and TNFAIP. Then the SRCC was calculated to assess co-expressed strength of each interaction between DEGs, based on which we constructed the co-expressed network to vividly exhibit their interlinking relationship with the Cytoscape software. The C4orf48 and SCN9A with the highest degrees were screened out from the sub-network drawn with the threshold of edges weight values > 0.8. The last but not least, the MF algorithm was used to extend the GBA principle based on neighbor voting, including the nearest-neighbor voting and indirect connections, and the ranking of genes arisen from MF scores. Further, a 3-fold cross validation was conducted to evaluate the accuracy of the algorithm, and as a result, the AUCs for the majority of functional terms were over 0.5, implying that the performance of GO terms was good, and we could use the AUCs to predict GO category membership. Six GO terms with the AUCs over 0.7 including the ion binding function and cellular response to stimulus process were identified and naturally regarded as the optional gene functions in EOPE.

The expression of abnormal genes is a critical factor in the onset and development of disease (13, 14). Therefore, it is very important for us to explore how the abnormal genes regulate the molecular function and biological processes associated with EOPE. In this study, the ion binding function was found to be most relevant to EOPE. During pregnancy, the placenta constitutes the main barrier for the exchange of material between mother and fetus, which becomes a vital organ that maintains nutrition and development of fetal. There are many ion channels in syncytiotrophoblast of human placenta, including chloride channels, calcium channels, sodium channels and potassium channels (15,16). The expression level of human calcium transport proteins CaT1 in the placenta is related to the absorptive amount of calcium ions and was time-dependent (17). Thus, these channels are closely related to the physiological function regulation of the placenta, and the change of ion binding function may influence the placental microenvironment, which was an important factor that lead to maternal hypertension and less nutrition to fetal.

Cellular response to stimulus is a defensive or adaptive response from the cells when they encounter harmful stimuli or lie in a hostile environment. The harmful stimuli includes reactive oxygen species (ROS), physical and chemical agents. The antioxidant reaction of the body can lead to the increase of lipid peroxides, and affect some cell signaling transduction and enzyme pathways related to the synthesis of vascularized substances (18). In our study, the cellular response to stimulus process was predicted an important function involved in the pathophysiology of preeclampsia by DEGs network-based GBA method. Through the literatures, we found the oxidative stress, one of the cellular response to stimulus processes, was reported elevated in mildly EOPE patients (19, 20). Besides, the oxidative stress was observed in the oocytes of obese animals before pregnancy. Therefore, the cellular response to stimulus function plays an important role in the occurrence and development of pathological EOPE. Based on the above mechanism, supplemental antioxidants in pregnancy may fight against oxidative stress, and prevent or delay the occurrence of EOPE.

## Conclusion

This study plays a great role for the molecular mechanisms underlying EOPE, and it may help us to understand the pathogenesis to focus on the genes which belong to these functions.

## Ethical considerations

Ethical issues (Including plagiarism, informed consent, misconduct, data fabrication and/or falsification, double publication and/or submission, redundancy, etc.) have been completely observed by the authors.
